# Insulin Modulates Inflammatory Cytokine Release in Acute Stages and Augments Expression of Adhesion Molecules and Leukocytes in Lungs on Chronic Stages of Paracoccidioidomycosis

**DOI:** 10.3389/fimmu.2020.583385

**Published:** 2020-11-18

**Authors:** Felipe Beccaria Casagrande, Sabrina de Souza Ferreira, Emanuella Sarmento Alho de Sousa, João Pedro Tôrres Guimarães, Lavínia Maria Dal’Mas Romera, Fernando Henrique Galvão Tessaro, Sandro Rogério de Almeida, Stephen Fernandes de Paula Rodrigues, Joilson O. Martins

**Affiliations:** ^1^ Laboratory of Immunoendocrinology, Department of Clinical and Toxicological Analyses, School of Pharmaceutical Sciences of University of São Paulo (FCF/USP), São Paulo, Brazil; ^2^ Laboratory of Mycology, Department of Clinical and Toxicological Analyses, School of Pharmaceutical Sciences of University of São Paulo (FCF/USP), São Paulo, Brazil; ^3^ Laboratory of Vascular Nanopharmacology, Department of Pharmacology, Institute of Biomedical Sciences, University of São Paulo (ICB/USP), São Paulo, Brazil

**Keywords:** macrophages, type 1 diabetes, inflammation, Paracoccidioidomycosis, systemic mycosis, fungal infection, vascular cell adhesion molecule-1 expression

## Abstract

Type 1 *diabetes* *mellitus* (T1D) is caused by partial destruction of the insulin-producing beta cells in the pancreas and is a major issue for public health care worldwide. Reduced or impaired immunological responses, which render patients more susceptible to infections, have been observed in T1D, and this dysfunction is often related to a lack of insulin in the blood. Paracoccidioidomycosis is an important systemic mycosis endemic in Latin America. To evaluate the effects of T1D on this fungal infection and the modulatory effects of insulin, we induced diabetes in C57Bl/6 male mice (alloxan, 60 mg/kg), infected the mice (Pb18, 1 x 10^6^ cells), and treated the mice with neutral protamine Hagedorn (NPH) insulin (2 IU/600 mg/dL blood glucose). Twenty-four hours after infection, infected diabetic mice showed reduced secretion of interferon (IFN)-γ and interleukine (IL)-12 p70 compared to infected nondiabetic controls. On the 45th day of infection, infected diabetic mice presented higher IFN-γ levels, a higher tumor necrosis factor (TNF)-α:IL-10 ratio, and lower adhesion molecule expression levels than nondiabetic mice. In the *in vitro* experiments, alveolar macrophages from diabetic animals showed reduced phagocytic activity compared to those from control animals at 4, 12, and 24 h. In infected diabetic mice, treatment with insulin restored IL-12 p70 levels at 24 h of infection, reduced IFN-γ levels and the TNF-α:IL-10 ratio at 45 days, and restored vascular cell adhesion molecule (VCAM)-1 expression in pulmonary blood vessels, and this treatment reduced the diminished phosphorylation of extracellular signal-regulated kinases (ERK) and increased nuclear factor-kappa-B(iκb)-α and jun amino-terminal kinases (JNK) p46 levels in infected nondiabetic mice. In addition, insulin promoted increased phagocytic activity in the alveolar macrophages of diabetic mice. These data suggest that T1D mice are more susceptible to Pb18 infection and that insulin modulates this inflammation in diabetic mice by augmenting the expression of adhesion molecules and leukocytes in the lungs and by reducing chronic inflammation.

## Introduction

*Diabetes mellitus* comprises a group of metabolic disorders characterized by a relative lack and/or reduced response to endogenous insulin on target cells (1), leading to metabolic and vascular complications related to hyperglycemia that affect several organs and systems (1, 2). It is estimated to affect more than 425 million patients worldwide (1, 2). Type 1 *diabetes mellitus* (T1D) is described as the partial or complete destruction of insulin-producing beta cells from Langerhans islets in the pancreas, and this causes hyperglycemia.

Among complications related to T1D, the impairment of immunological responses to diverse inflammatory stimuli has been widely observed in diabetic patients, and this includes reduced production of the inflammatory cytokine interleukin (IL)-1β in *Mycobacterium tuberculosis* infection (3) and increased susceptibility to skin, bone, and joint infections and to fungal diseases (4). Some of the impairments found in people with diabetes are reproduced in animal models of T1D, such as reduced expression of intercellular adhesion molecule (ICAM)-1, reduced migration of leukocytes to inflammatory sites, diminished secretion of tumor necrosis factor (TNF)-α (5), reduced phagocytic activity of diabetic rat neutrophils (6), and reduced bactericidal activity of Paneth cells in diabetic mice (7). Experimental treatment with insulin was observed to promote different outcomes regarding immunological responses under different inflammatory conditions (4–7). However, there are still few studies relating insulin to the chronic pattern of inflammation observed in some fungal infections.

Paracoccidioidomycosis (PCM) is a systemic granulomatous disease caused by *Paracoccidioides* sp. fungi; PCM was first observed in 1908 and described in 1930 (8), and it ranks as the top cause of hospitalizations among systemic mycoses (9). PCM occurs when a host inhales the mycelial form of the fungus originating from the soil, and several variables determine the severity of the disease, such as the inhaled fungal load, strain virulence, and host immunocompetence (9). As occurs in many systemic mycoses, phagocytosis and destruction of infectious agents by cells of the immune system is highly important for the efficient and asymptomatic resolution of PCM infection (10). An efficiently developed or preexisting T helper 1 (Th1) response and the secretion of cytokines that activate phagocytic activity on macrophages and stimulate T cells, interferon (IFN)-γ, IL-12, and TNF-α are often observed to contain the infection during the subclinical period (11–13). On the other hand, a deficient or inhibited Th1 response is observed in individuals in which PCM has progressed to the chronic stage, suggesting higher susceptibility to the agent (11, 14).

T1D is an important disease that has a significant socioeconomic impact worldwide; it is ranked among the main public health issues (1, 2). At a smaller scope, hospitalizations and mortalities resulting from decompensated PCM have a high occurrence in Latin America, especially in Brazil (15). Although the association between T1D and PCM in literature is not strong, both are considered highly undiagnosed/underreported diseases (1, 8), and our studies show an important rise in susceptibility for a more severe case of PCM in T1D mice, possibly resulting from an overall reduced protection against the pathological agent (16), and that insulin partially restored cellular inflammation indexes. In this work, we aimed to study the effects of insulin on leukocyte presence and activity in a mouse model of T1D infected with *P. brasiliensis*.

## Methods

### Animals

This study used pathogen-free mice of the C57Bl/6 strain, and it was performed in accordance with the guidelines accepted by the Brazilian National Council for Control of Animal Experimentation (CONCEA) of the School of Pharmaceutical Sciences (FCF) in the University of São Paulo (USP) (the project is registered under the permit CEUA/FCF/512). Surgical procedures were performed under anesthesia (ketamine hydrochloride 90 mg/kg and xylazine hydrochloride 10 mg/kg, respectively; Sespo, Brazil), and all care was taken in order to minimize animal suffering. This work used 49 mice, distributed into 3–6 animals/group, all male and weighing 18–22 g in the beginning of the experimental period, in which they were maintained in a controlled environment at 22°C and a 12-h light-dark cycle. Chow and water were available ad libitum throughout the experiment.

### Diabetes Mellitus

An alloxan-induced model was used to induce T1D in mice (16, 17). Briefly, mice received intravenous injections of alloxan monohydrate [60 mg/kg of animal dissolved in 100 µL of sterile saline solution (NaCl 0.9%); Sigma-Aldrich, United Kingdom]. Mice from control groups were injected with 100 µL of sterile saline instead. The parameters confirming diabetic state were obtained 10 days after the injection by measuring blood glucose (Accu-Chek Advantage II, Roche Diagnóstica, São Paulo, Brazil) in samples collected from mouse tails. Animals presenting blood glucose higher than 300 mg/dL were considered diabetic.

### Paracoccidioides brasiliensis

An isolate of a *Paracoccidioides brasiliensis* strain known to be virulent (16, 18) (Pb18; Laboratory of Mycology, School of Pharmaceutical Sciences, University of São Paulo, Brazil) was used for this study. The cultures were grown in Sabouraud’s semisolid medium at 37°C with weekly subculture. Colonies of Pb18 yeasts were collected in sterile phosphate buffer solution (PBS; 18 mM Na_2_HPO_4_, 3 mM NaH_2_PO_4_H_2_O, and 140 mMNaCl in Mili-Q water) and vortexed for 1 min before filtration with a 40 µm cell strainer (BD Biosciences) thrice. The concentration of yeasts was counted in a Neubauer hemocytometer and adjusted to a standard before inoculation.

### Pb18 Infection

On the 10th day, after confirmation of T1D, mice were anesthetized (ketamine/xylazine hydrochloride) and, upon unconsciousness being confirmed, were inoculated with 1 x 10^6^ yeast cells in 50 µL sterile PBS *via* intratracheal injection. Noninfected groups received 50 µL sterile PBS instead by the same procedure.

### Insulin Treatment

**Figure 1:**
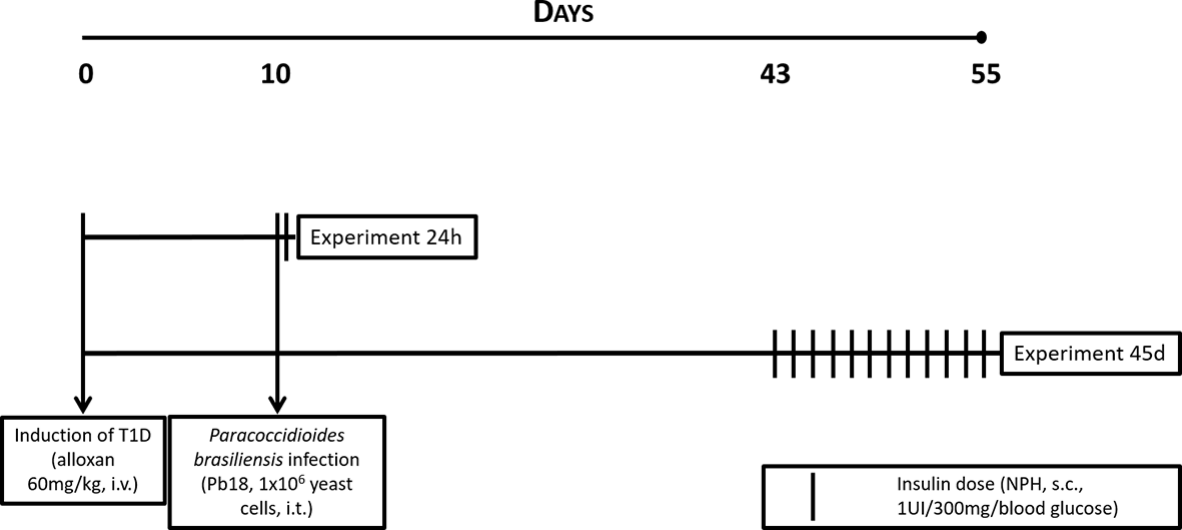
Experimental protocol for T1D, infection with *P. brasiliensis*, and treatment with NPH insulin in mice.

Eight hours after infection by Pb18, mice from the insulin-treated groups received neutral protamine Hagedorn insulin (NPH; Eli Lilly, São Paulo) by subcutaneous injections for evaluation of its effects on a 24-h infection. Alternatively, 33 days after infection, mice received one injection a day at 6 pm during the 12 days preceding the experiment, completing a 45-day infection. In both cases, dose was calculated according to blood glucose levels presented by mice (2 IU insulin/600 mg/dL of blood glucose) (16, 17). A graphical schema for the protocol can be observed in **Figure 1**.

### Organs Harvesting and Cell Count

After euthanasia 24 h or 45 days following infection, lungs were harvested and washed in sterile PBS and then disrupted manually in PBS using a Potter-Elvehjem tissue grinder. The large particulate material was removed by filtration with a 70-µm cell strainer, and the filtrate was centrifuged. Supernatant was stored in -80°C for posterior analysis while cells in the pellet were suspended in PBS supplemented with 3% fetal bovine serum (FBS). The leukocyte concentration of each sample was assessed using Neubauer hemocytometer slides and Turk’s solution, and results were presented in cells/mL.

### Quantification of cytokines

IFN-γ, IL-10, TNF-α, and IL-12 p70 were quantified in lung macerate supernatant with the use of a Cytometric Bead Array Mouse Inflammation Kit (BD, Biosciences). Assays were performed according to the manufacturer’s instructions, and samples were measured by flow cytometer (FACSCanto II, BD Biosciences), where results were analyzed using BD CBA Software (BD, Biosciences). Results are expressed in pg/mL.

### RNA Extraction and Real-Time PCR

Total RNA from lung homogenates was extracted following the protocol described in Cold Spring Harbor Protocols (19). cDNA was synthesized with RevertAid First Strand cDNA Synthesis Kit (lot 00376305, ThermoFisher Scientific) and real-time PCR was executed with the following primers: *il12, tnfα, il6, il10, il4*, and *tgfβ* (all from Exxtend^®^; More information about the primers can be found in the **Supplementary Material**) at Applied Biosystems *StepOnePlus*™ Real-Time PCR System (ThermoFisher Scientific). Relative gene expression was calculated by comparative threshold cycle (Ct) and expressed relative to the controls (ΔΔCt method).

### Quantification of Signaling Molecules *via* Western Blot

Lungs of mice were harvested after 45 days of Pb18 infection and disrupted with RIPA lysis buffer. The homogenates had their protein determined (Pierce BCA Protein Assay Kit; Thermo Fisher Scientific Inc., IL) and calculated into 50-µg samples. Proteins were separated *via* electrophoresis in polyacrylamide gel and then transferred to nitrocellulose membranes (Amersham Biosciences Corp., NJ, USA). Following 1 h blocking (5% nonfat dried milk in Tris-buffered saline Tween (20 mM Tris, 150 mM NaCl, 1% Tween 20; TBST), membranes were washed with TBST thrice and stayed overnight at 4°C in primary antibodies against the target molecules (p38 MAPK, P-p38 MAPK, ERK 1/2 MAPK, P-ERK 1/2 MAPK, JNK, iκB-α, TLR-2, pAKT, PKC-α). All antibodies were purchased from Cell Signaling Biotechnology (MA, USA). After 3 washing steps, membranes were incubated for 1 h with antirabbit secondary antibody (1:10,000; Abcam). β-actin (1:50,000, 1 h; Sigma) was used as loading control. More information about the antibodies can be found in the **Supplementary Material**. For development, we used chemiluminescence detection in an Amersham Imager 680 blot and gel imager (Amersham, Buckinghamshire, UK), and densitometric analysis of the bands was performed using Image Studio software (LI-COR Biosciences, Lincoln, Nebraska, USA). Results are represented by densities of each band divided by density of its respective loading control.

### Quantification of Adhesion Molecule

For this study, we quantified expression of VCAM-1 by the immunohistochemistry assays. Briefly, the superior right lobes of the lungs were stored in 10% formaldehyde and dehydrated by baths in crescent concentrations of ethanol (70% to 100%) before being imbedded in paraffin. Transversal sections were collected in silanized slides and fixated with formaldehyde. In the moment of analysis, slides are bathed on xylene twice for 5 min, the sections are hydrated in baths in decreasing concentrations of ethanol (twice in 100%, then 95% and 80%), 3 min each. Antigen retrieval, blocking of peroxidases, and blocking for unspecific binding were performed using reagents and instructions provided by the kit EnVision FLEX+ (Dako, Denmark), followed by overnight incubation with primary antibody (1:50; Santa Cruz Biotechnology) at 4°C in a humid chamber. After washings, sections were incubated for 1 h with a secondary antibody (conjugated to horseradish peroxidase) provided by the kit and, following a washing step, incubated with substract containing 3,3-diaminobenzidine for 8 min. Slides were washed and mounted with organic resin (Entellan, MERK) and then observed in a light microscope Leica DMLFS (Leica Microsystems, Wetzlar, Germany), using a DFC300FX camera (Leica Microsystems) to capture the images.

Five pictures showing at least one blood vessel were taken from each sample, and quantification of the mean values of staining per area was performed with ImageJ software.

### Infection Index and Phagocytosis CFU

Alveolar macrophages (AM) were obtained by bronchoalveolar ex vivo lavage from diabetic (alloxan 60 mg/kg in NaOH 0.9%; 10 days) and nondiabetic (NaOH 0.9%; 10 days) mice. The fluid was centrifuged at 259 G/10 min and the cells in the pellet suspended in RPMI-1640 (Gibco) medium. Volumes containing 5 x 10^5^ AM were transferred to each well containing glass cover slides, maintained in an incubator at 37°C and 5% CO_2_ for 1 h for adhesion, and then washed with warm PBS and incubated with RPMI-1640 supplemented with 2% FBS (Vitrocell, São Paulo, Brazil) for 18 h.

To evaluate the phagocytic activity, nonadhered cells were washed out with warm PBS, and the adhered cells were incubated (RPMI-1640. 10% FBS, penicillin 100 µg/mL, gentamicin 100 µg/mL, and streptomycin 100 µg/mL) in the presence or absence of insulin [insulin from bovine pancreas, reconstituted and prepared according to manufacturer’s instructions (0.005%) Sigma-Aldrich; Catalog number i6634]. Volumes containing 5 x 10^5^ yeasts of Pb18 were added to the wells, followed by incubation for 4, 12, or 24 h in a CO_2_ incubator to allow adhesion and ingestion of the fungus. After incubation, cover slides were washed and stained with hematoxylin and eosin and then mounted on microscopy slides with organic resin and observed in a light microscope. Each well had 300 macrophages analyzed, and the infection index was calculated following the formula (II = Internalized Yeast/Total Macrophages*100) (20).

Assays of CFU were performed to quantify the yeast internalized by the macrophages. AM cultures (RPMI-1640, 10% FBS, penicillin 100 µg/mL, gentamicin 100 µg/mL, and streptomycin 100 µg/mL) with or without insulin (insulin from bovine pancreas, 0.005% Sigma) were infected with Pb18 yeasts (5 x 10^5^ yeasts) in sterile PBS and incubated (37°C, 5% CO_2_). After 4, 12, or 24 h of interaction, medium was removed, and wells were washed three times to remove nonadhered yeasts. Macrophages were ruptured using 200 µL 0.1% Triton (Sigma-Aldrich, USA) in cold PBS. The cell lysate was plated on brain hearth infusion (BHI; KASVI) semisolid medium and incubated for 20 days at 37°C, after which the recovered colonies were counted.

### Data Analysis

The data were evaluated by analysis of variance (ANOVA) followed by the Tukey-Kramer posttest for multiple comparison using GraphPad Prism 7.0 software. Data are here represented by mean values ± standard error mean (SEM), and values *p*<0.05 were considered significant for this study.

## Results

### Characterization of T1D Model

In our previous publication, we showed that the injection of alloxan caused a significant reduction in weight gain (mean ± SEM; control, 2.1 ± 0.4 g in 10 days and 6.8 ± 0.8 g in 55 days; diabetic, 0.5 ± 0.3 g in 10 days and 3.1 ± 1.5 g in 55 days; *p*<0.001) and sharply elevated blood glucose levels (control, 195 ± 9 mg/dL in 10 days and 167 ± 5%nbsp;mg/dL in 55 days; diabetic, 489 ± 18 mg/dL in 10 days and 506 ± 66 mg/dL in 55 days; *p*<0.001) (16).

### Evaluation of Inflammation in the Lungs at Early Stages of PCM (24 h)

In our previous publication, we showed that the injection of alloxan caused a significant reduction in weight gain (mean ± SEM; control, 2.1 ± 0.4 g in 10 days and 6.8 ± 0.8 g in 55 days; diabetic, 0.5 ± 0.3 g in 10 days and 3.1 ± 1.5 g in 55 days; *p*<0.001) and sharply elevated blood glucose levels (control, 195 ± 9 mg/dL in 10 days and 167 ± 5 mg/dL in 55 days; diabetic, 489 ± 18 mg/dL in 10 days and 506 ± 66 mg/dL in 55 days; *p*<0.001)

**Figure 2:**
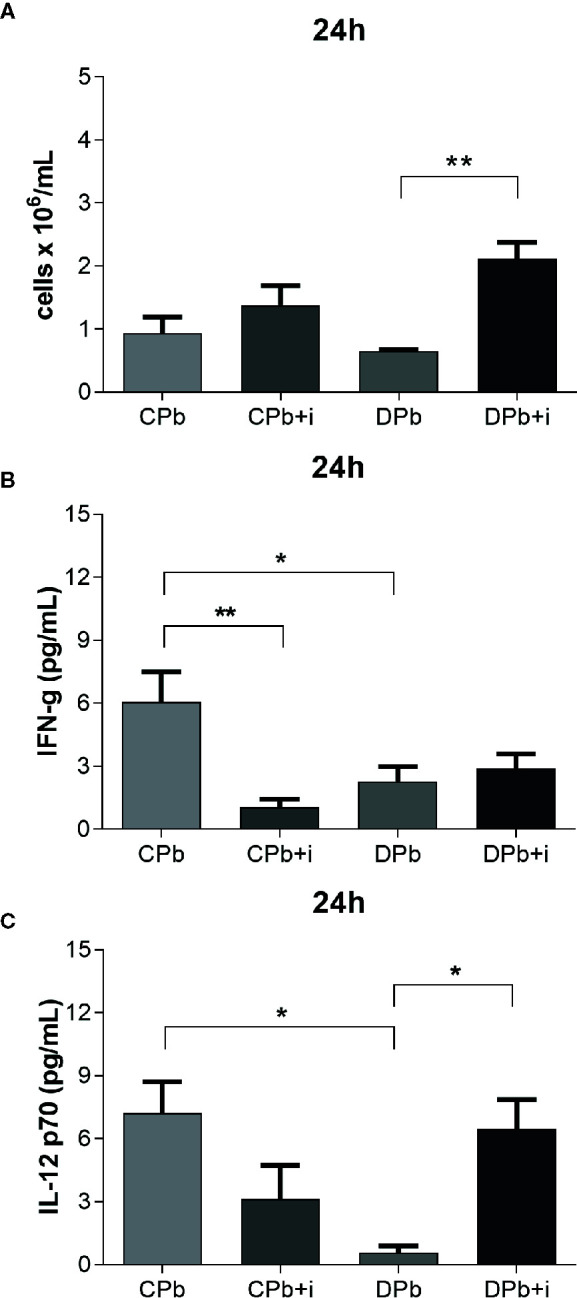
Assessment of inflammation in lungs – 24 h. T1D mice and control mice infected with Pb18 yeasts with and without one dose of insulin 8 h after infection. Samples were obtained 24 h after infection. **(A)** Leukocyte count in lungs; **(B)** IFN-γ in lungs; **(C)** IL-12 p70 in lungs. Bars represent mean value ± SEM. *p < 0.05; **p < 0.01.

In the analysis of samples obtained 24 h after infection, we observed no significant difference between diabetic and nondiabetic mice in the leukocyte count in the lungs (**Figure 2A**). Compared to infected control mice, infected diabetic mice showed reduced levels of the proinflammatory cytokines IFN-γ and IL-12 p70 (**Figures 2B, C**; *p*=0.0223 and *p*=0.0337, respectively).

Treatment with a single dose of insulin resulted in a significant 3-fold increase in leukocytes in the lungs as well as a more than 10-fold increase in secreted IL-12 p70 levels in diabetic animals (**Figures 2A, C**; *p*=0.0026 and *p*=0.0426, respectively). Interestingly, insulin also promoted a reduction in the levels of IFN-γ in infected nondiabetic mice (**Figure 2B**).

**Figure 3:**
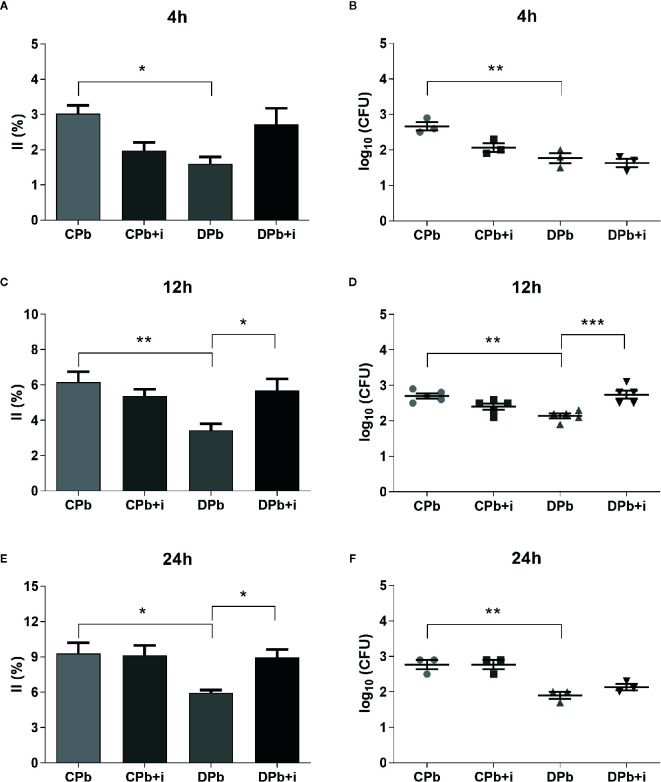
Phagocytic activity *in vitro*. AM from T1D mice and control mice were infected with Pb18 yeasts *in vitro* with or without insulin in the medium. **(A, B)** 4 h after infection; **(C, D)** 12 h after infection; **(E, F)** 24 h after infection. Bars represent mean value ± SEM. II, infection index; CFU, colony forming units. *p < 0.05; **p < 0.01; ***p < 0.001.

AM were obtained from nondiabetic and diabetic mice and then infected *in vitro* with Pb18 yeast to evaluate their infection capacity in the presence or absence of insulin. The growth of colony forming units (CFUs) on cell lysates of the same samples was analyzed to confirm the internalization of the agent. **Figure 3** shows that AM from diabetic animals presented a reduced infection index compared to cells from nondiabetic mice. This difference was observed at 4 h (*p*=0.0306; **Figure 3A**), 12 h (*p*=0.0071; **Figure 3C**), and 24 h (*p*=0.0236; **Figure 3E**). Based on our CFU analysis, the cell lysate of macrophages from nondiabetic mice presented an increased number of colonies compared to that of AM from diabetic mice after 4, 12, and 24 h of interaction (**Figures 3B, D, F**).

The addition of insulin to the culture medium did not change AM from nondiabetic mice but restored phagocytic capacity in AM from diabetic mice in experiments at 12 h (1.7-fold; *p*=0.0222) and 24 h (1.5-fold; *p*=0.0452). This increase in yeast internalized by AM from diabetic animals in the presence of insulin was significant at 12 h interaction (**Figure 3D**).

### Evaluation of Inflammation in the Lungs at Late Stages of PCM (45 Days)

**Figure 4:**
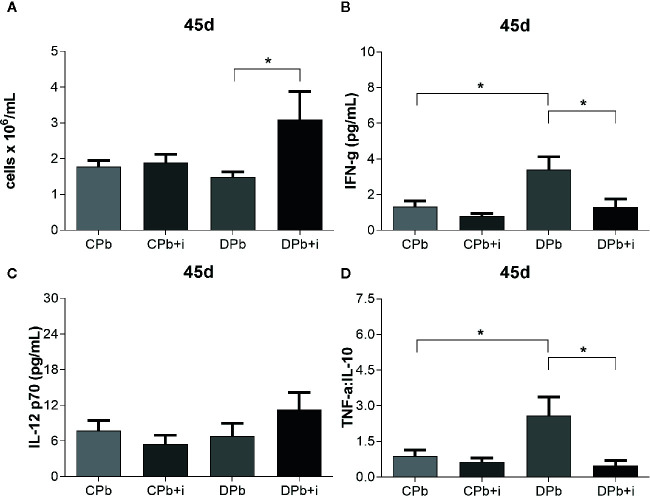
Assessment of inflammation in the lungs at 45 d. T1D mice and control mice infected with Pb18 yeasts with or without daily doses of insulin 12 days before the experiment. Samples were obtained 45 days after infection. **(A)** Leukocyte count in lungs; **(B)** IFN-γ in lungs; **(C)** IL-12 p70 in lungs. **(D)** TNF-α:IL-10 ratio. Bars represent mean value ± SEM. *p < 0.05.

Obtained at later stages of the infection, 45-day samples showed no difference in the number of leucocytes present in the lungs of diabetic and nondiabetic mice (**Figure 4A**). Diabetic mice showed a more proinflammatory environment than nondiabetic mice as evidenced by higher IFN-γ levels and a higher TNF-α:IL-10 ratio (**Figures 4B, D**; *p*=0.0240 and *p*=0.0356, respectively), whereas the levels of IL-12 p70 did not differ between the groups (**Figure 4C**). Twelve injections of insulin given once a day before the experiment did not affect nondiabetic mice in regard to these parameters although, in diabetic animals, this treatment increased leukocytes (**Figure 4A**; *p*=0.0183). In addition, insulin treatment reduced IFN-γ levels (2.6-fold; *p*=0.0463) and the TNF-α:IL-10 ratio (4.4-fold; *p*=0.0276) in diabetic mice (**Figures 4B, D**).

**Figure 5:**
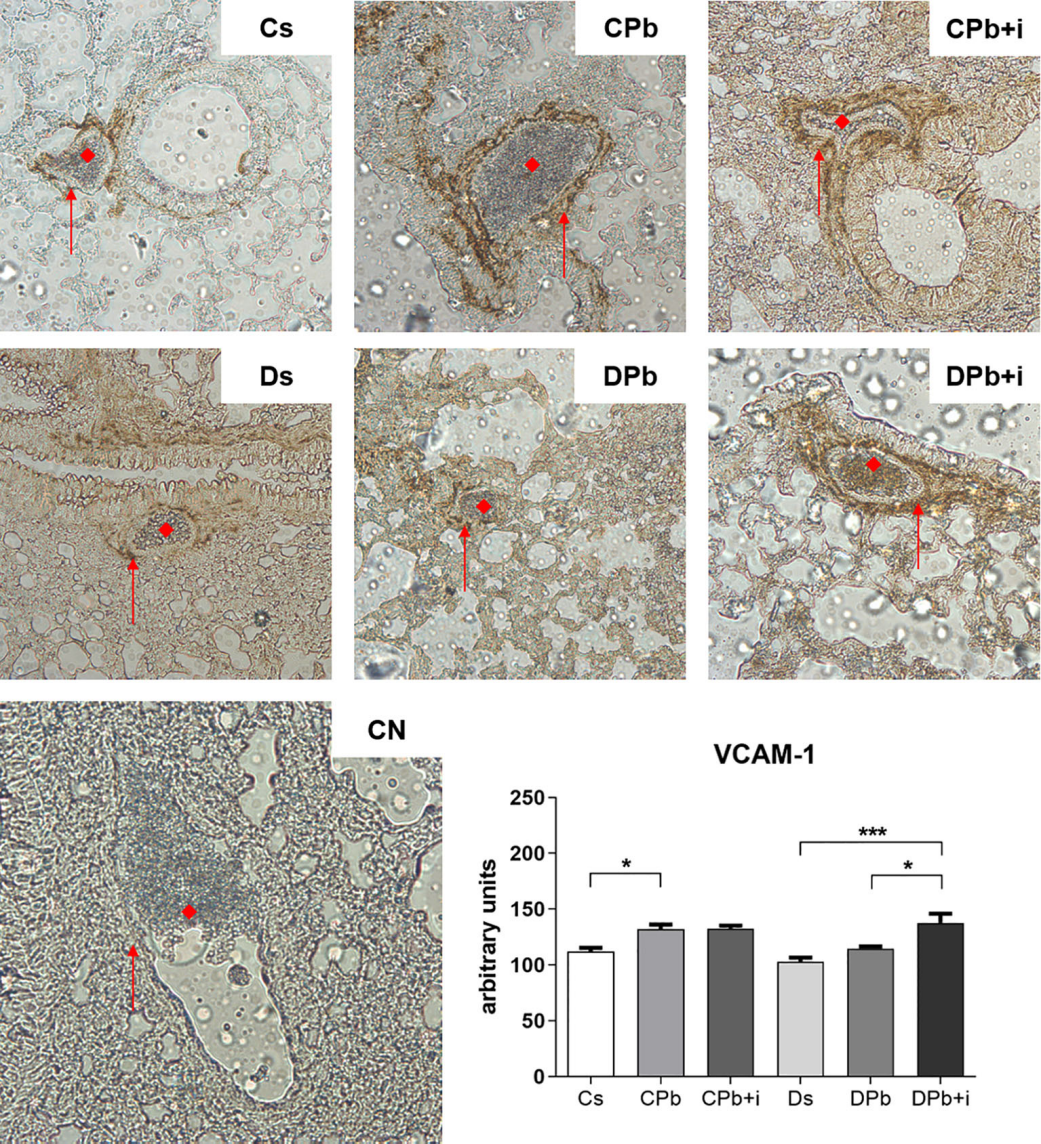
Expression of VCAM-1 in the pulmonary vascular wall. T1D mice and control mice infected with Pb18 yeasts with or without daily doses of insulin 12 days before the experiment. Samples were obtained 45 days after infection. Immunostaining is represented by brown coloration on the vascular wall and is shown by arrows. (◆) Pulmonary blood vessel (magnification, x20). Bars represent mean value ± SEM. *p < 0.05; ***p < 0.001.

Blood vessels in lungs harvested after 45 days of infection were stained to quantify the presence of vascular cell adhesion molecule (VCAM)-1 *via* immunohistochemistry. **Figure 5** shows a significant increase in the expression of VCAM-1 in the vessels of nondiabetic infected mice (*p*=0.0205) but not in diabetic mice. Treatment with insulin did not change VCAM-1 expression in infected nondiabetic animals, but increased the expression of this adhesion molecule in diabetic mice (**Figure 5**; *p*=0.0211).

**Figure 6:**
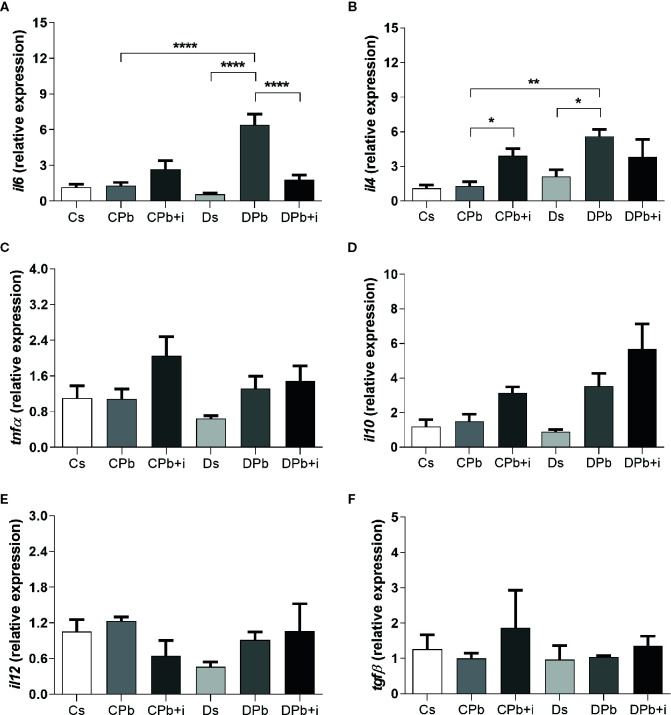
Gene expression in lungs of T1D mice and control mice after 45 days of infection with Pb18 yeasts with and without daily doses of insulin 12 days before the experiment. Expression of **(A)** *il6*; **(B)** *il4*; **(C)** *tnfα*; **(D)** *il10*; **(E)** *il12*; and **(F)** *tgfβ*. Bars represent the mean value ± SEM. *p < 0.05; **p < 0.01; ****p < 0.0001.

**Figure 6** shows the results of RT-PCR analysis performed on lungs after 45 days of infection. Compared to infected nondiabetic mice, diabetic mice infected with Pb18 presented higher *il6* and *il4* expression (**Figures 6A, B**; *p*<0.0001 and *p*=0.0026, respectively). Treatment with insulin resulted in reduced *il6* expression in infected diabetic mice (**Figure 6A**; *p*<0.0001) and increased *il4* expression in infected nondiabetic mice (**Figure 6B**; *p*=0.0389). There were no significant differences in the expression of *tnfα*, *il10*, *il12*, and *tgfβ* between diabetic and nondiabetic mice with or without insulin treatment.

**Figure 7:**
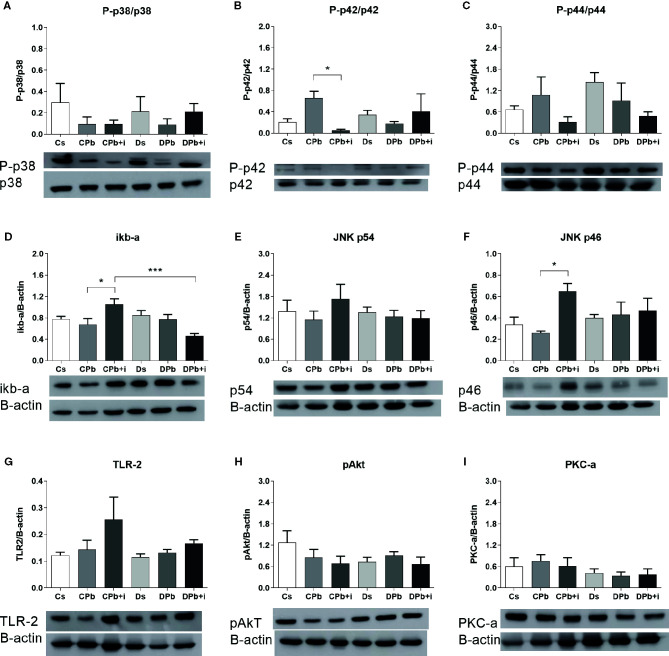
Quantification of signaling molecules in the lungs of T1D mice and control mice after 45 days of infection with Pb18 yeasts, with or without daily doses of insulin 12 days before the experiment. Levels of **(A)** P-p38; **(B)** P-p42 ERK; **(C)** P-p44 ERK; **(D)** iκB-α; **(E)** p54 JNK; **(F)** p46 JNK; **(G)** TLR-2; **(H)** pAkt; and **(I)** PKC-α. Bars represent the mean value ± SEM. *p < 0.05; ***p < 0.001.

We evaluated the levels of signaling molecules in inflammatory pathways in the lungs *via* Western blot analysis. In samples obtained 45 days after infection, the concentrations of the studied molecules in infected and noninfected mice were not different for both the diabetic and nondiabetic groups. Treatment with insulin, however, resulted in diminished phosphorylation of ERK in infected nondiabetic mice as seen by a decrease in the p-ERK:total ERK ratio (**Figure 7B**; *p*=0.0460). Insulin treatment also resulted in augmented levels of whole fractions of iκb-α (**Figure 7D**; *p*=0.0496) and JNK p46 (**Figure 7F**; *p*=0.0320) in infected nondiabetic mice but did not induce changes in infected diabetic mice. This experimental model did not result in significant alterations in the ratios of p-p38:total p38 and p-p44:total p44 (**Figures 7A, C**, respectively) or in the levels of JNK p54, TLR-2, p-Akt, and PKC-α (**Figures 7E–I**, respectively).

## Discussion

The data shown in this study are summarized in **Table 1** and suggest the involvement of insulin in the inflammatory process in mice infected with *P. brasiliensis*, which is a dimorphic fungus endemic in Latin America. Henceforth, we used a model of the relative absence of insulin, alloxan-induced T1D, combined with exogenous insulin treatment and *in vitro* insulin treatment. Previous studies from our group have established a protocol of insulin treatment of alloxan-induced diabetic animals in which the effective dose of insulin able to significantly reverse inflammatory parameters observed in diabetic animals was chosen. Even though this dose only partially reduces the blood glucose, elevated levels of insulin can be observed during the whole time of the experiment (16, 21). Although this dose was able to restore inflammatory parameters in diabetic mice, it was not sufficient to return glucose levels in diabetics to normal values, and it also had no effect on mortality. Thus, we can associate the effects in insulin-treated mice primarily to the presence of insulin rather than to reduction of glycemia.

**Table 1:** Summarized data results observed in nondiabetic and diabetic mice infected with Pb18, with and without treatment with insulin.

Effects of Pb18 infection in diabetic mice with and without insulin treatment		
	T1D	T1D + Insulin
**24 hours – lungs**		
Leukocytes number	NS	Higher
IFN-γ levels	Lower	NS
IL-12 p70 levels	Lower	Higher
**Infection Index – alveolar macrophages**		
4 h	Reduced	NS
12 h	Reduced	Augmented
24 h	Reduced	Augmented
**45 days – lungs**		
Leukocytes number	NS	Higher
IFN-γ levels	Higher	Lower
IL-12 p70 levels	NS	NS
TNF-α:IL-10 ratio	Higher	Lower
*Il4* relative expression.	Higher	Lower
*Il6* relative expression	Higher	NS
VCAM-1 expression	NS	Higher

Comparisons were made versus their respective control (infected nondiabetic mice for infected diabetic mice and diabetic nontreated mice for diabetic treated mice).

Deleterious effects of T1D on the immunological system have been observed before, and studies have associated it with the relative absence of insulin rather than hyperglycemia onset (22, 23). Anjos-Valotta et al. (5) associated T1D with reduced expression of TNF-α and ICAM-1 in TNF-stimulated diabetic rats, and treatment with insulin restored these parameters (5). On the other hand, treatment with insulin has also been related to a decrease in inflammation in diabetic patient as well as the suppression of NF-κB expression and molecules whose gene transcription is dependent on NF-κB, such as monocyte chemotactic protein-1 and -9 and plasminogen activator inhibitor-1 (23, 24). Although most studies in the field consistently agree that T1D causes a higher susceptibility to different types of infections, the modulatory role of insulin seems to differ according to the inflammatory stimulus to which the subject is exposed (3, 5, 25).

For this study, we chose experimental *P. brasiliensis* infection as an inflammatory stimulus due to its epidemiological and socioeconomic relevance to further elucidate the effects of T1D and insulin treatment. Animal models of PCM have been used to better understand the mechanisms and symptoms of PCM, and we chose a mouse strain previously described to have intermediate susceptibility to Pb18 (26).

An effective inflammatory response to Pb18 relies on both innate and adaptive immunity: phagocytosis and destruction of the etiological agent; production and secretion of Th1 proinflammatory cytokines, such as TNF-α, IFN-γ, and IL-12; followed by activation of macrophages, TCD4+ cells, and TCD8+ cells. When the inflammatory response is efficient, the infection is often contained in the subclinical stage (12, 13, 27). In this work, in samples obtained 24 h after infection, we observed less acute inflammation in diabetic animals than in nondiabetic animals with lower levels of IFN-γ and IL-12 p70 in the lungs. In 2001, Benard et al. observed that more severe PCM in an animal model was related to reduced levels of IFN-γ and IL-12, and Kashino et al. (27) associated low levels of IFN-γ with the faster development of PCM in susceptible strains of mice (27). Here, infected diabetic mice treated with insulin presented higher number of leukocytes in the lungs and higher secretion of IL-12 p70 in the lungs, suggesting a more effective response to Pb18 infection.

Phagocytosis and the destruction of pathological agents are crucial to the host’s response to several different infections, especially during acute inflammation; these mechanisms represent the main protective mechanisms against most systemic mycosis (28), including PCM, and effective responses leading to phagocytosis and destruction of the yeast cells have been related to faster subclinical clearance and better prognosis (10, 12). The role of macrophages and how they are activated in response to a pathogen are key elements of an appropriate immune response, and the metabolic environment has an important impact on this response. Previous studies showed that lineage (29) and peritoneal macrophages (30) cultured under high glucose conditions tend to present M2-like phenotype characteristics, which would polarize them toward an adaptive rather than an innate immune response, and other studies relate the impaired phagocytic ability in AM of diabetic rats to reduced phosphorylation of ERK, Akt, and PKC-δ resulting from deficient bonding of leukotrienes to FcγR signaling pathways (31). In addition, Ayala et al. show that high glucose levels appear to modify macrophage behavior, affecting different aspects of diabetic (impaired phagocytic ability, reduced production of reactive hydrogen species, and reduced expressions of TLR-4 on the cell surface) and healthy bone marrow-derived macrophages under the same LPS stimulus, hypothesizing that hyperglycemia leaves a glucose legacy, altering the basal steady state of macrophages (32). More recently, Tessaro et al. (33) also showed that *in vitro* treatment with insulin is able to amplify inflammatory cytokine secretion by bone marrow-derived macrophages from diabetic mice stimulated with LPS by enhancing phosphorylation of MAPK (p42 MAPK, p44 MAPK, p46 SAPK, p54 SAPK) resulting from TLR-4 activation with LPS, and mice deficient of mechanisms related to detection and phagocytosis, such as expressions of TLR-2 and TLR-4, were observed to be more susceptible to PCM (10). In the results of this work, macrophages obtained from T1D mice showed reduced phagocytic activity against Pb18 yeast cells compared to AM obtained from controls, and the presence of insulin in the medium restored phagocytic activity even though insulin treatment *in vivo* in diabetic animals did not alter the levels of IFN-γ. These results could help to explain the high susceptibility presented by T1D mice to Pb18 observed in previous studies (16). These data also corroborate previous findings in animal models as macrophages from hyperglycemic mice presented reduced phagocytic activity against *Salmonella typhimurium* (34), and diabetic rat neutrophils were also less efficient against *Candida albicans* yeasts (35). These phagocytic dysfunctions in T1D were associated with changes in cell metabolism and in insulin levels. Alba-Loureiro and collaborators’ study in 2006 observed that insulin treatment restored phagocytic function in diabetic rat neutrophils (6), whereas Yano et al. (36) observed that treatment with insulin augmented the phagocytic activity of T1D mouse neutrophils against *Staphylococcus aureus* and increased bactericidal capacity. These results provide supporting evidence that a metabolic change caused by insulin could directly affect susceptibility to infections related to T1D.

Moreover, insulin seems to influence in multifaceted ways macrophages from distinct origins. In alloxan-induced diabetes in rats without infection, hyperglycemia and absence of insulin did not change, for example, autophagosome LC3 levels in bronchoalveolar lavage fluid (37). When bone marrow cells were differentiated into M1-like macrophages, those cells derived from diabetic animals without infection have lowered their autophagosome LC3 content. Conversely, cells differentiated into M2-like macrophages have their autophagic LC3 content enhanced. When one of the proteins (Atg12) responsible for conjugating LC3 into the phagosome was screened, it was diminished in the splenic macrophages from red pulp of the diabetic animals without infection compared to healthy control. When diabetic rats were treated with insulin, splenic macrophages failed to restore Atg 12 content (37). In addition, the modulatory effects of insulin in chronic inflammations in experimental models of T1D has also been reported. In previous studies using similar models of later stages of PCM in T1D mice, we observed diabetic mice to be more susceptible to PCM than nondiabetic mice, probably due to a reduction in populations of TCD4+ cells, TCD8+ cells, NK cells, and B lymphocytes, which are reportedly important to contain the spread and proliferation of the etiological agent (38, 39). Even though the absolute number of leukocytes in bronchoalveolar lavage fluids did not vary within the groups, the reduction of these populations resulted in augmented fungal loads in the lungs of diabetic mice compared to the lungs of controls (16). In the present work, samples obtained after 45 days of infection revealed that diabetic mice still presented signs of strong ongoing inflammation, characterized by high IFN-γ levels and a high TNF-α:IL-10 ratio, whereas this inflammation was found to be subsided in the nondiabetic groups.

The regulation of genes responsible for inflammatory conditions by insulin has been observed in other studies (23, 24) even in the presence of T1D. We observed higher *il6* expression in T1D mice infected with Pb18 than in noninfected T1D mice, and treatment with insulin seemed to decrease the expression of this gene. Another interesting finding was that i*l4* gene expression was higher in T1D mice infected with Pb18 than in noninfected T1D mice. The progression of PCM is associated with high IL-4 production (40). Although a Th1 immune response appears to effectively control infection, the fungus itself is able to modulate metabolite production and surface molecule interactions with immune cells to produce both pro- and anti-inflammatory cytokines, which makes it difficult for the body to develop resistance during infection (41). In this regard, studies show that an impaired immune response in diabetes facilitates the establishment of infection (22), which may justify the dichotomy observed in the relative gene expression of both pro- and anti-inflammatory cytokines but may not reflect the secreted protein scenario. To better understand the effect of insulin on the inflammatory focus, we analyzed signaling pathways in lung homogenate. Proteins in the mitogen-activated protein kinase (MAPK) signaling pathway have important roles in the activation and differentiation of T lymphocytes (42). Studies suggest that ERK phosphorylation is related to T cell polarization to a Th2 profile (43), and p38 phosphorylation is associated with Th1 polarization (44). Viardot and associates showed in 2007 that insulin promoted the differentiation of T cells to a Th2 profile, reducing the Th1 cell proportion and the IFN-γ:IL-4 ratio. These results were associated with increased ERK phosphorylation and reduced p38 phosphorylation (45). In this study, the results observed in the lungs of mice 45 days after Pb18 infection showed that insulin treatment resulted in diminished phosphorylation of ERK in nondiabetic mice, and p-p38 was unaltered. Other results, such as increased IL-4 expression and reduced IFN-γ in this group, oppose a diminished response of the Th2 profile.

The importance of cell migration to the inflammatory site, thus allowing the development of an efficient immunological response, has been evidenced by previous studies showing the role of proinflammatory cytokines and adhesion molecules (6, 46, 47). Studies also show that cell migration through the endothelium is dysregulated in T1D patients, and this is often related to alterations on the expressions of adhesion molecules. Sharma et al. (48) observed that T1D patients with retinopathy had accentuated levels of ICAM-1 compared to controls, and diabetic patients with chronic kidney disease showed increased VCAM-1, both suggesting more severe inflammation in patients with pathologies related to diabetes (48, 49). On the other hand, in animal models of T1D, diabetic rats showed reduced inflammation in addition to lower levels of ICAM-1 compared to nondiabetic rats in response to external inflammatory stimuli (6). In this work, different than infected nondiabetic controls, infected diabetic mice did not show higher expressions of VCAM-1 in the lungs, and although we did not perform a cell migration assay, both the expressions of VCAM-1 in pulmonary vessels and the number of leukocytes in this organ were restored after treatment with insulin. Interestingly, this augmentation of leukocyte numbers was found accompanied by a reduction of IFN-γ levels and TNF-α:IL-10 ratio, suggesting a reduced Th1 inflammation and perhaps involvement of other groups of cytokines that were not addressed by this study. Other than IFN-γ and TNF-α, the presence of IL-6 and IL-23 have been associated to formation and maturation of granulomas (50). Moreover, in their work *in vitro*, Calich and Kashino showed in 1998 that secretions of IL-5 by lymphocytes of mice was associated to susceptibility to PCM when it happened during both earlier and later stages of infection and to resistance when observed only during the later stages of it (51), suggesting that the priming of leukocytes to a Th2-type profile increases susceptibility only when it occurs on early stages of PCM, and that may be more important than the population of leukocytes in the inflammation site, the polarization of the type of cytokines produced by them have direct impact on resistance/susceptibility to PCM.

The clinical relevance of T1D, its increasing incidence worldwide and its impact on health care and on social and economic fields are widely known, and studies to better elucidate its deleterious effects and indirect consequences are important. In conclusion, our work showed an impaired acute response to Pb18 in mice with alloxan-induced diabetes characterized by reduced IFN-γ and IL-12 p70 levels, decreased phagocytic activity, and chronic inflammation 45 days after the infection. Moreover, we suggest that insulin modulates this response, as it restored IL-12 p70 levels during acute inflammation and increased presence of leukocytes in inflammatory sites, leading to a reduction in chronic inflammation in T1D mice.

## Data Availability Statement

The raw data supporting the conclusions of this article will be made available by the authors, without undue reservation.

## Ethics Statement

The animal study was reviewed and approved by Brazilian National Council for Control of Animal Experimentation (CONCEA) of the School of Pharmaceutical Sciences (FCF), in the University of São Paulo (USP) (the project is registered under the permit CEUA/FCF/512).

## Author Contributions 

FC and JM elaborated the project and conceived the experiments. FC, SF, FT, LR, JG, and ES conducted the experiments. JM, SA, and SR contributed with reagents, materials, analysis, and expertise. FC and JM wrote this paper and all authors reviewed the manuscript. All authors contributed to the article and approved the submitted version.

## Funding

The authors are supported by grant 2014/05214-1, 2017/11540-7 and 2020/13215-9 from São Paulo Research Foundation (FAPESP), grant 470523/2013-1 and 301617/2016-3 from National Counsel of Technological and Scientific Development (CNPq, Projeto Universal 2013, PQ-1D) and Coordenação de Aperfeiçoamento de Pessoal de Nível Superior (CAPES).

## Conflict of Interest

The authors declare that the research was conducted in the absence of any commercial or financial relationships that could be construed as a potential conflict of interest.
