# Supplementary material for: Insulin Modulates Inflammatory Cytokine Release in Acute Stages and Augments Expression of Adhesion Molecules and Leukocytes in Lungs on Chronic Stages of Paracoccidioidomycosis
Source: Front Immunol. 2020 Nov 18;11:583385. doi: 10.3389/fimmu.2020.583385 (PMC7708333; doi:10.3389/fimmu.2020.583385)
Supplement: Supplementary file 1 [file DataSheet_1.docx]

**Insulin modulates inflammatory cytokines release on acute stages and augments expression of adhesion molecule and leukocytes in lungs on chronic stages of Paracoccidioidomycosis**

Felipe Beccaria Casagrande, Sabrina de Souza Ferreira, Emanuella Sarmento Alho de Sousa, João Pedro Tôrres Guimarães, Lavínia Maria Dal’Mas Romera, Fernando Henrique Galvão Tessaro, Sandro Rogério de Almeida, Stephen Fernandes de Paula Rodrigues, Joilson O. Martins*

**Supplementary figure 1-** Referring to the item 2.8 of the manuscript, a table with the primers used for RTPCR in this study.

| **Gene** | **Sequence** |
| --- | --- |
| il6 | 5’TAGTCCTTCCTACCCCAATTTCC3’ and  5’TTGGTCCTTAGCCACTCCTTC3’ |
| il10 | 5’GCTCTTACTGACTGGCATGAG3’ and  5’CGCAGCTCTAGGAGCATGTG3’ |
| il4 | 5’GGTCTCAACCCCCAGCTAGT3’ and  5’GCCGATGATCTCTCTCAAGTGAT3’ |
| stat1 | 5’GACTTCAGACACAGAAATCAACTC3’ and  5’TTGACAAAGACCACGCCTT3’ |
| tgfB | 5’ATTGCTTCAGCTCCACACAG3’ and 5’TGTTGGTTGTAGAGGGCAAG3’ |
| tnfa | 5’CCCTCACACTCAGATCATCTTCT3’ and 5’GCTACGACGTGGGCTACAG3’ |
| il12 | 5’TGGTTTGCCATCGTTTTGCTG3’ and 5’ACAGGTGAGGTTCACTGTTTCT3’ |
| hprt | 5’AGCAGGTCAGCAAAGAACT3’ and 5’CCTCATGGACTGATTATGGACA3’. |

**Supplementary figure 2-** Referring to the item 2.9 and 2.10, follows a table with the antibodies used in this study.

| **Antibody** | **Company (#Catalog)** | **Dilution** |
| --- | --- | --- |
| PKC-α-β | Cell Signaling (#2056) | 1:1000 in TBS, 5% BSA, 0,1% Tween20 |
| p38 MAPK | Cell Signaling (#9212) | 1:1000 in TBS, 5% BSA, 0,1% Tween20 |
| p-p38 MAPK | Cell Signaling (#4511) | 1:1000 in TBS, 5% BSA, 0,1% Tween20 |
| ERK1/2 (p42/p44) | Cell Signaling (#9102) | 1:1000 in TBS, 5% BSA, 0,1% Tween20 |
| p-ERK ½ | Cell Signaling (#9101) | 1:1000 in TBS, 5% BSA, 0,1% Tween20 |
| iκB-α | Cell Signaling (#9242) | 1:1000 in TBS, 5% BSA, 0,1% Tween20 |
| pAkt (Sr473) | Cell Signaling (#4060) | 1:1000 in TBS, 5% BSA, 0,1% Tween20 |
| JNK | Cell Signaling (#9252) | 1:1000 in TBS, 5% BSA, 0,1% Tween20 |
| TLR-2 | Cell Signaling (#13744) | 1:1000 in TBS, 5% non-fat dry milk, 0,1% Tween20 |
| VCAM-1 | Santa Cruz Biotech (#sc-8304) | 1:50 in TBS, 5% BSA, 0,1% Tween20 |
| Anti-Rabbit HRP | Sigma-Aldrich (#A0545) | 1:80000 in TBS, 5% non-fat dry milk, 0,1% Tween20 |
| Β-actina | Sigma-Aldrich (#A5316) | 1:40000 in TBS, 5% non-fat dry milk, 0,1% Tween20 |
| GAPDH | Sigma-Aldrich (#G9545) | 1:50000 in TBS, 5% non-fat dry milk, 0,1% Tween20 |
